# Healthy Communities for Youth: A Cost Analysis of a Community-Level Program to Prevent Youth Violence

**DOI:** 10.1007/s11121-024-01729-z

**Published:** 2024-09-30

**Authors:** Zhongzhe Pan, Derek A. Chapman, Terri N. Sullivan, Diane L. Bishop, April D. Kimmel

**Affiliations:** 1 School of Public Health, Department of Health Policy, Virginia Commonwealth University, Richmond, VA USA; 2 Center On Society and Health, Virginia Commonwealth University, Richmond, VA USA; 3 School of Public Health, Department of Epidemiology, Virginia Commonwealth University, Richmond, VA USA; 4 Clark Hill Institute for Positive Youth Development, Virginia Commonwealth University, Richmond, VA USA; 5 College of Humanities and Sciences, Department of Psychology, Virginia Commonwealth University, Richmond, VA USA

**Keywords:** Cost analysis, Youth violence, Community-based intervention, Implementation science

## Abstract

Youth violence is a national public health concern in USA, especially in resource-constrained urban communities. Between 2018 and 2021, the Healthy Communities for Youth (HCFY) program addressed youth violence prevention in select economically marginalized urban communities, with the HCFY program reducing the likelihood of youth-involved violent crime. Leveraging costs from program expense reports, this study analyzes the costs of the HCFY program in order to inform policymaking and the program’s future ongoing implementation. Total HCFY program costs were $821,000 ($290,100 annually including program start-up costs) over the 34-month project period. Operationalization costs contributed the largest share (64.8%), with 45% attributable to intervention coordinators. In the intervention community, the program costs $100 per capita, $1100 per youth-involved crime case, and $8100 per youth-involved violent crime case. Findings were sensitive to the number of youth-involved crime or violent crime cases and costs of high-level program leadership and self-evaluation analysts, with the per youth-involved violent crime case cost ranging between $700 and $1600 over the program period. Analysis of HCFY program costs is an important step in determining the affordability of a community-level program to prevent youth violence in resource-limited urban communities.

## Introduction

Youth violence remains a national public health concern in the United States. Homicide is the second leading cause of death among 15- to 24-year-olds nationally and has been the leading cause of death among Black males for over two decades (Centers for Disease Control Prevention, 2021), with violence exposure among youth and families costing nearly $215 billion annually (Centers for Disease Control Prevention, 2021). Youth violence occurs in not only larger urban cities but also in mid-sized cities that may have fewer financial resources. In both settings, resource distribution overall and for youth violence is inequitable, particularly for Black youth and families who live in economically marginalized urban communities and shoulder the burden of violence exposure (Nation et al., 2021).

The current study focuses on youth violence in a mid-sized urban community in the Southeastern United States where youth and young adult homicide rates are 7 times the national average (Bishop et al., 2020; Centers for Disease Control Prevention, 2021). Through a system of complementary community-level interventions, Healthy Communities for Youth (HCFY) addressed youth violence prevention by increasing the opportunity structure for positive youth development in select neighborhood communities with concentrated poverty and high community rates of violence due to long-standing systemic racism and disinvestment. Over the intervention period (April 2019–June 2021), the likelihood of youth-involved violent crime decreased in the intervention community by 39% compared to the control community and by 92% compared to the remainder of the city outside the HCFY study areas, although these decreases were not significant using a quasi-experimental difference-in-difference analysis.

Cost analysis of community-level programs like HCFY plays a key role in informing health policy and practice. The approach is an analytic process in which elements of a program are identified, the amount of each element used is quantified, and a cost is applied to each element (Drummond et al., 2015). Together, this information is evaluated and used not only to summarize overall program costs but also to examine a program’s key cost drivers. Cost analysis serves as the foundation for multiple types of economic evaluations—e.g., cost-effectiveness analysis, budget impact analysis, budget optimization, and return on investment—all of which can inform budget allocations when resources are limited (Crowley et al., 2018; Drummond et al., 2015). Importantly, the approach is used by policymakers to inform program affordability, sustainability, and scalability (Crowley et al., 2018), where variation in the valuation of different program elements and/or the amount used inform the feasibility of program adoption in the real world.

Cost analysis of systems of community-based youth violence prevention programs is limited. More common are cost analyses of single-intervention community-level programs. For example, Kuklinski and colleagues (2012) assessed costs for the Communities that Care youth violence prevention model, one of the complementary interventions in HCFY’s system of interventions. Similarly, Rice et al. (2024) examined the overall costs of individual community-level teen and youth violence prevention strategies to gain insight into the economic impact on local health department budgets of individually implementing multiple youth violence prevention programs, with one of the evaluated programs, the Safe Street Program, also reported separately (Webster et al., 2023). While variations in the program target population, setting (geographic, public health practice vs research), youth violence model, and costing approach will contribute to cost differences across individual programs, a nuanced understanding of program costs for integrated and complementary youth violence prevention models remains understudied.

The goal of the current study is to conduct an economic assessment of HCFY, a system of community-level youth violence prevention strategies, to inform the feasibility and sustainability of future program implementation in an urban, economically marginalized community.

## Methods

### Healthy Communities for Youth (HCFY)

Focused on two low-income neighborhood communities in Richmond, Virginia, HCFY combined two complementary community intervention strategies to (1) prevent youth violence, defined as young people aged 10–24 years purposely exerting “physical force or power to threaten or harm others” (David-Ferdon et al., 2014) and (2) to focus on social connectedness and building opportunities for youth to enhance their strengths and foster positive relationships. One strategy, the Communities that Care (CTC) prevention system, has five phases that center on working with a coalition to select relevant prevention strategies to reduce community rates of youth violence (Clark-Hill Institute for Positive Youth Development, 2023; Hawkins et al., 2014).

For phase 1 of CTC, we collaborated with an existing youth violence prevention coalition, the Inspire Workgroup, with HCFY leadership and intervention staff serving on the Workgroup throughout the project. In phase 2, a smaller coalition, the Community Intervention Team (CIT), was created and focused specifically on intervention strategies for Community A.

The implementation of CTC phases 3 and 4 spanned December 2018 to April 2019. During these phases, HCFY leadership, intervention, and data management staff provided ongoing coordination and support for CIT meetings (e.g., meeting logistics/preparation and monitoring progress) and also with the collection, analysis, and presentation of data. The data manager provided ongoing support for all data collection efforts and managed all aspects of the ongoing collection of the surveillance data. As part of phases 3 and 4, members of the CIT reviewed (1) community survey data, including risk and protective factors related to youth violence and its prevention and positive youth development opportunities in Community A that were provided by youth (ages 12–17) caregiver pairs and young adults (18–22), (2) qualitative data provided by community members and partners addressing community strengths, resource needs, priorities for mobilization, and biggest concerns related to youth violence, and (3) a resource and gaps assessment. Using these data, the CIT created an action plan that included selected intervention strategies. The intervention strategies represented in the CIT action plan included (a) ongoing coordination with youth- and family-serving organizations in Community A to increase PYD opportunities; (b) collaboration between the HCFY intervention and leadership staff, CIT, and Inspire Workgroup to align community resources; (c) intergenerational family meetings with outcomes designed to promote positive interactions and social connectedness (e.g., community events such as bike rides, Black History Month celebration, and game nights); and (d) the Walker-Talker and Plain Talk (WT-PT) community engagement model.

The WT-PT community engagement model focused on building community awareness of resources that enhance youths’ safety and positive youth development (e.g., summer camp and after-school life-skill training) and removing some barriers to accessing these resources (Allison et al., 2011; Douglas, 1998). The WT-PT model, based on the Annie E. Casey “Plain Talk” model, encompassed (a) messages about positive parenting and parenting strengths created by caregivers and community partners, (b) PT conversations ranging from one-on-one to small group conversations to large community conversations to discuss and share parenting strengths and practices and issues related to youth safety and positive development, and (c) direct outreach to youth and families to build community awareness of resources that enhance youths’ safety and positive youth development (e.g., summer camp and after-school life-skill training), provide self-care resources, and remove some barriers to accessing these resources (Allison et al., 2011; Douglas, 1998). CTC phase 5 involved evaluating the impact of the HCFY prevention strategies on community rates of youth violence.

### Study Sample and Timeline

HCFY was implemented in two neighborhood communities, A and B, with around 5000–6000 population and similar risk profiles for youth violence between October 2018 and July 2021. Phases 1–4 of CTC were implemented between October 2018 and April 2019, while community prevention strategies and phase 5 of CTC were implemented together with the WT-PT community engagement model from April 2019 to July 2021. The intervention was not examined in Community B because the program was implemented in this community solely during the COVID-19 pandemic. Community-level effects of HCFY were evaluated using a generalized additive model comparing the likelihood of youth-involved violent incidents in the intervention neighborhood community (Community A) to a control neighborhood community with similar socio-demographic characteristics (Community C) and to the rest of the city, excluding Communities A, B, and C prior to and during the study period. The likelihood of youth-involved violent crime decreased at the end of the intervention in Community A by 39% compared to Community C (OR = 0.93; 95% confidence interval (CI): 0.63, 1.39 before the intervention; OR = 0.57; CI: 0.37, 0.90 after the intervention) and by 92% compared to the rest of the city (OR = 1.28; CI: 0.98, 1.68 before the intervention; OR = 1.02; CI: 0.74, 1.40 after the intervention). These decreases were not significant in a subsequent quasi-experimental difference-in-difference analysis.

### Data

HCFY program cost data were collected at the community level and came from monthly program expense reports, with the data extracted retrospectively. These data represented costs for the intervention only (i.e., Community A); we assumed no costs for the HCFY control community (i.e., Community C), as the control community did not receive the intervention. We did not include costs for the intervention conducted in Community B because the effect of the HCFY intervention was not assessed. We emphasize that data were available at the community level only and not at the individual level. Thus, unit costs and resource utilization, which are often used in individual-level (versus community-level) cost analysis, were not available.

HCFY cost data for Community A included.

1. Rent and supplies associated with community and WT conversations, as well as related family intergenerational gatherings, and
2. Salaries and fringe benefits for leadership overseeing implementation, project coordinators for overall coordination, intervention coordinators responsible for intervention implementation, communication specialists responsible for designing public-facing program materials, WTs involved in neighborhood outreach, and other program staff for community resource assessment and program evaluation.

Costs of donated space and supplies, as well as the opportunity cost of a volunteer advisory board of community stakeholders, were not included. However, the inclusion of these costs was assessed in the sensitivity analysis. No research, only staff or other research-related costs were included (Gold et al., 2022). Opportunity costs reflecting, for example, productivity gains or costs of averted morbidity and mortality due to crime prevention were not included (Anderson, 2021). These opportunity costs were not assessed in this analysis due to a lack of individual-level data on time spent participating in the intervention and on improvement in health status due to crime prevention.

Data also included reported crime incidents involving 10- to 24-year-olds (victims or offenders), 2018–2021, from the city’s police department. Reported crimes were dichotomized as violent (e.g., murder, manslaughter, assault, robbery) and non-violent (all other reported crimes) (Federal Bureau of Investigation, 2018; Masho et al., 2019). Crimes were spatially categorized into Community A, Community C, or not in a study community if their geocoded incident address fell within a given community’s boundaries. Reported crimes were not included if their incident address was incomplete, missing, or located outside the city (< 1% of all incidents).

### Cost Categorization

We applied a top-down costing approach to assign and apportion the costs of resources used by the HCFY program based on the time when costs were incurred and the responsibilities of staff and other program personnel. Community-level HCFY program costs were categorized as start-up (October 2018–March 2019), operationalization (April 2019–July 2021), and continuous self-evaluation (October 2018–July 2021). *Start-up costs* were costs for designing and developing the intervention’s community action plan. These costs included prorated annual salaries and fringe benefits paid to high-level program leadership overseeing program implementation and intervention coordinators to manage initial intervention activities, as well as independent contractor fees paid to communication specialists responsible for designing public-facing program materials. *Operationalization costs* were costs for putting the HCFY intervention into practice and included the costs of facilities and supplies for community events and activities, independent contractor fees for communication specialists, hourly salaries for WTs, and prorated annual salaries and fringe benefits for central administration personnel, i.e., high-level program leadership and intervention coordinators responsible for managing all aspects of intervention implementation. Finally, *continuous self-evaluation costs* were costs for assessing community awareness of and capacity for implementing the community action plan, as well as continuous self-assessment (i.e., neighborhood- and community-level data curation across sectors, ongoing process- and outcome-based evaluation for continuous adaptation and improvement) (Kuklinski et al., 2012). Specifically, continuous self-evaluation costs included salaries and fringe benefits for high-level program leadership, intervention coordinators, and data analysts for data curation and ongoing self-assessment, as well as tuition and stipends for graduate students or other analytic support.

We distinguish continuous self-evaluation costs (Kuklinski et al., 2012), which are integral to the program’s ongoing self-assessment to improve implementation, from strictly research-related costs, which represent resources used to collect and analyze program data above and beyond continuous self-evaluation. No research-related costs were included in this analysis. While fixed costs for research and development costs are recommended for inclusion in some economic analyses (Neumann et al., 2016), we note that analogous costs are represented as start-up costs in the current analysis.

We made several assumptions to apportion personnel costs. First, we assumed costs related to high-level program leadership were uniformly distributed across the start-up, operationalization, and continuous self-evaluation categories. Second, we assumed salaries, fringe benefits, and independent contractor fees for intervention coordinators and communication specialists were distributed equally between the start-up and implementation cost categories; they were not distributed to the evaluation category, given these personnel did not engage in community awareness and resource capacity assessment or in continuous self-assessment. Third, we assumed that annual salaries and fringe benefits were paid monthly with the distribution of 2019 salaries and fringe benefits categorized as start-up and operationalization costs based on the length of each category within the calendar year.

### Cost Analysis

We assessed the economic costs of the HCFY program from a payer perspective because this study is intended to provide information that supports budgeting decisions made by policy decision-makers. We calculated the cost of the program intervention, overall and for each cost category (start-up, operationalization, continuous self-evaluation) over the 34-month program period and for each calendar year. Across the program period and for each calendar year, we also estimated annual HCFY costs per total population, cost per reported crime involving youth, and cost per reported violent crime involving youth, all for Community A.

Costs were inflation-adjusted using the US Consumer Price Index and reported in constant 2021 US dollars (Bureau of Labor Statistics, 2024). Final cost estimates were rounded to the nearest hundred dollars to reflect uncertainty in reporting (Gold, 1996).

### Sensitivity Analysis

We conducted one-way sensitivity analyses varying, separately, key elements related to reported crime cases, personnel costs, and non-personnel costs. For reported crime cases, we varied the reported number of crime cases and violent crime cases by + / − 25% to capture variation in youth violence in different communities (Ramsey et al., 2015) and variation in crime reporting (Thompson & Tapp, 2022). For personnel costs, we varied the salaries paid to WTs by + / − 25% to account for position turnover and variation in WT working hours. We also accounted for variation in high-level program leadership, continuous self-evaluation analysts, and stakeholder advisory board needs by removing salaries for high-level program leadership or continuous self-assessment support (lower bound), while in separate analysis additionally incorporating the costs of honoraria (conservatively, $500 per board member per year) for a 10-member advisory board (upper bound), which was served by community volunteers in the HCFY program. Finally, for non-personnel costs, we varied the costs of rent and supplies by applying a 25% reduction in rent and supplies (lower bound) to capture changes in market prices, while in separate analysis additionally including continuous, prorated rent and supply costs over the entire project period (upper bound) to reflect more typical operationalization of HCFY than occurred during the COVID-19 public health emergency, when PT conversations and family intergenerational gatherings were held virtually instead of in person.

We additionally conducted a multi-way sensitivity analysis to capture best- and worst-case cost scenarios. The best-case scenario assumed minimum HCFY program costs by applying a 25% increase in the number of reported crime cases and violent crime cases, no high-level program leadership or continuous self-assessment support, and a 25% decrease in rent and supplies. The worst-case scenario assumed maximum HCFY program costs by applying a 25% decrease in the number of reported crime cases and violent crime cases, honoraria for a 10-member advisory board, and continuous, prorated rent and supply costs over the project period.

## Results

The HCFY intervention cost a total of $821,900 over the 34-month project period, with a mean annual cost of $290,100 (Table 1). Operationalization comprised the largest share of program costs at 64.8%, followed by continuous self-evaluation (22.5%) and start-up (12.7%) costs. Costs for intervention coordination were highest during start-up and operationalization, accounting for 58% of the total start-up and 45% of total operationalization costs, respectively. WTs involved in neighborhood outreach represented the second highest (23%) of total operationalization cost. Data analytic support contributed the largest share (45%) to continuous self-evaluation costs. High-level program leadership and intervention coordination costs were substantial across all cost categories, accounting for 25% and 36% of total HCFY costs, respectively.

**Table 1:** Economic costs of the Healthy Communities for Youth (HCFY) program in a neighborhood community in Richmond, Virginia^a^

Cost category	Economic costs (2021 US$)				
	**2018**^**b**^	**2019**	**2020**	**2021**^**b**^	**Total**
Start-up					
Leadership^c^	23,000	20,700	-	-	43,700
Intervention coordination^d^	28,800	31,500	-	-	60,200
Communication^e^	300	-	-	-	300
Sub-total	52,100	52,200	-	-	104,300
Operationalization					
Leadership^c^	-	62,200	24,600	7500	94,300
Intervention coordination^d^	-	96,100	94,000	47,700	237,800
Communication^e^	-	12,200	29,100	5400	46,700
Walker-talkers	12,700	51,100	40,500	16,400	120,700
Other^f^	-	16,000	9600	7500	33,100
Sub-total	12,700	237,600	197,900	84,400	532,600
Continuous self-evaluation					
Leadership^c^	11,500	41,500	12,300	3700	69,000
Data analytic support^h^	9700	40,600	22,500	11,000	83,800
Other analytic support^g^	2700	16,100	12,600	800	32,200
Sub-total	23,900	98,300	47,400	15,500	185,000
Total program cost	88,700	388,000	245,200	100,000	821,900
Average annual program cost	-	-	-	-	290,100

^a^Economic costs are reported in 2021 US dollars and rounded to two significant digits, where applicable. Costs with no specified usage in the raw data are not reported

^b^Costs accrued over a 3-month period (October–December) in 2018 and a 7-month period (January–July) in 2021

^c^Defined as the economic costs (annual full-time effort × annual salary + fringe benefits) of those managing and directing project activities. The economic costs for leadership are assumed to be allocated equally across the start-up, implementation, and evaluation processes

^d^Defined as the economic costs (hours worked × hourly wage + fringe benefits; annual full-time effort × annual salary + fringe benefits) of those managing and implementing the youth violence intervention

^e^Defined as the economic costs (hours worked × hourly wage + fringe benefits; annual full-time effort × annual salary + fringe benefits) of project-related communication, including communication coordination and graphic and web design

^f^Other costs represent non-personnel costs, including rental fees for community support space (e.g., churches) and supplies

^g^Defined as costs for analytic support include salaries paid to students who work for data analysis

^h^Data analytic support provided by the data manager included ongoing support for all data collection efforts and managed all aspects of the ongoing collection of the surveillance data

**Fig. 1:**
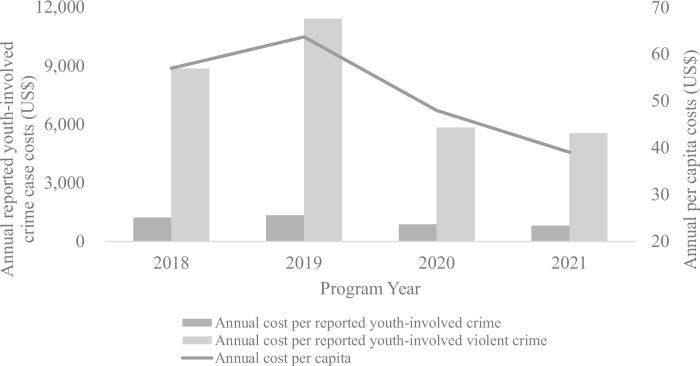
Annual HCFY program costs per youth-involved reported crime case, per reported youth-involved violent crime case, and per capita, 2018–2021

Over the 34-month project period, HCFY covered a mean population of 5810 adults and youth in Community A, where a total of 754 youth-involved crime cases and 106 youth-involved violent crime cases were reported. On average, and after accounting for uncertainty in the cost reporting, HCFY costs $100 per capita, $1100 per reported youth-involved crime case, and $8100 per reported youth-involved violent crime case (Fig. 1).

### Sensitivity Analysis

**Fig. 2:**
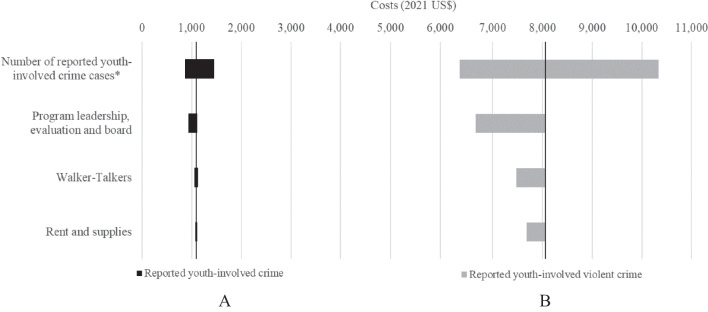
Sensitivity analyses to assess variation in HCFY program costs per reported crime case, by type of crime. Legend: Shown are sensitivity analysis results based on variation in the Healthy Communities for Youth (HCFY) program personnel and non-personnel expenses, Community A. Sensitivity analysis for reported youth-involved crime cases and reported youth-involved violent crime cases were varied by + / − 25%. Sensitivity analysis for program leadership, evaluation, and advising ranged from the exclusion of economic costs for high-level program leadership (i.e., faculty) and program evaluation (i.e., data analyst and other analytic support) to additionally including honoraria for advisory board members (assumed to be $500 per board member per year). Sensitivity analysis for implementation ranged from higher turnover (and associated higher costs) of project walker-talkers to budget constraints leading to fewer on-the-ground personnel; these scenarios were captured by varying walker-talker-associated costs + / − 25%. Finally, sensitivity analysis for non-personnel costs represented variation in the need for rental space and supplies, which was captured by a 25% reduction in cost for rents and supplies and the inclusion of prorated costs over the project period. The horizontal axis is the cost per reported youth-involved crime case (**A**) and cost per reported youth-involved violent crime case (**B**), while the vertical axis shows the sensitivity analyses assessed. The dark grey bars represent the range in costs per reported youth-involved crime case and cost per reported youth-involved violent crime case, respectively, associated with each sensitivity analysis. The vertical black line indicates the baseline cost per reported youth-involved crime case (**A**) and cost per reported youth-involved violent crime case (**B**). The key driver of costs per reported youth-involved crime case (**A**) and cost per reported youth-involved violent crime case (**B**) is the number of reported youth-involved crime cases and number of reported youth-involved violent crime cases, respectively

The number of reported youth-involved crime cases was the leading driver of HCFY intervention costs, followed by program personnel, which collectively comprised high-level program leadership, continuous self-evaluation consultants, and the stakeholder advisory board (Fig. 2). When we varied the number of any reported crime case by + / − 25%, HCFY costs per any reported youth-involved crime case ranged from $900 to $1500; we found similar wide variation for reported youth-involved violent crime cases, with HCFY program costs per reported youth-involved violent crime ranging from $6400 to $10,700. When high-level program leadership and continuous self-evaluation support were not included, HCFY costs $900 per any reported youth-involved crime case and $6900 per reported youth-involved violent crime case. Inclusion of annual honoraria for a 10-member advisory board had a more limited impact, increasing the cost per any reported youth-involved crime case and per reported youth-involved violent crime case by < 2.5% (+ $30 and + $200, respectively). Variation in WT costs (+ / − 25%), as well as variation in the cost of rent and supplies, had minimal impact, with less than a $40 difference (any reported youth-involved crime case) and less than a $300 difference (reported youth-involved violent crime case) in costs compared to baseline.

For best- and worst-case cost scenarios, we found substantial variation in HCFY program costs. In the best-case scenario, the average annual costs of HCFY decreased by 18.8% (to $235,600 annually), with a 36.4% decrease in costs per reported youth-involved crime case (to $700) and a 35.8% decrease in reported youth-involved violent crime case (to $5200). The worst-case scenario resulted in a 7.8% increase in average annual costs; program costs per reported youth-involved crime case and violent crime case increased by 45.4% and 43.2% to $1600 and $11,600, respectively.

## Discussion

Leveraging economic data from HCFY—a system of community-level youth violence prevention programs in a neighborhood community in Richmond, Virginia—we found that the program costs $290,100 annually, including start-up costs, with program operationalization representing the largest cost share. Program costs per reported youth-involved violent crime case were sensitive to the number of reported youth-involved violent crime cases and the inclusion of high-level program leadership.

Annual costs for HCFY’s collection of programs and prevention strategies for one neighborhood community are generally lower than costs allocated annually by urban city governments to implement single youth violence prevention programs, including those solely in an operationalization phase. For example, the city of Richmond, Virginia, allocated over $550,000 to the We Matter Community Violence Intervention and Prevention Initiative (We Matter CVIPI) for fiscal year 2024 (City of Richmond, 2023), with this initiative providing financial incentives and support (e.g., recreational activities, youth mentors, mental health providers) at three community sites reaching approximately 2800 youth in grades 6–12 in the community (United States News & World Ranking, 2024). The We Matter CVIPI program costs around $200 per capita, which is higher compared to the HCFY program ($100 per capita). We emphasize that the We Matter program has additionally received prior state-level support (Virginia Department of Criminal Justice Services, 2023), and thus these additional funds likely do not support start-up costs. Further, also for fiscal year 2024, the city of Richmond allocated approximately $400,000 to the community-wide Youth Gun Violence Prevention Initiative for youth at risk of engaging in gun violence (City of Richmond, 2023). While limited information is available on the population reached, this cost is less comparable with the annual costs of the HCFY program. Future studies are warranted to further inform resource allocation to adopt the HCFY program in the community. These studies include cost-effectiveness analysis (assesses the economic efficiency of the HCFY program compared to existing violence prevention program(s)), budget impact analysis (examines the financial consequences of integrating the HCFY program in violence prevention efforts), and other decision-making processes (e.g., alternative competing priorities, priority criteria, multicriteria decision-making, stakeholder analysis) at the community level.

HCFY program costs of $8100 per reported youth-involved violent crime case likely serve as a conservative upper bound estimate. Indeed, evidence suggests substantial underreporting of violent crimes, with 52% to as high as 93.1% of violent crimes unreported to law enforcement (Thompson & Tapp, 2022; Wu et al., 2019). Reports indicate routine misclassification of violent crimes as more minor offenses, with, for example, undercounting of aggravated assault by 36% due to misclassification as a simple assault (Poston & Rubin, 2014). For the current analysis, a conservative 25% increase in the number of reported youth-involved violent crime cases decreased HCFY costs by $1700 per reported youth-involved violent crime case. These decreases would be even greater if underreporting and misclassification of violent crimes occurred in the city at the levels reported in the literature.

Scaling up HCFY to other urban or suburban neighborhoods with relatively high rates of youth-involved violence has the potential for economies of scale. For HCFY, “economies of scale” refers to decreases in average annual program costs and costs per reported youth-involved violent crime case for a new neighborhood community with similar rates of youth-involved violence, given that infrastructure could be shared across neighborhood communities (Johns & Torres, 2005). That is, the average annual cost for a new neighborhood using shared infrastructure resources within the existing HCFY program will cost less than if the program were implemented separately and independent of the existing program. The concept of economies of scale is particularly relevant in urban environments where neighborhood communities interact and share resources (Johns & Torres, 2005). For example, resources that support central administration, including high-level program leaders and intervention coordinators, can be simultaneously applied to multiple communities. Other infrastructure, such as the development and maintenance of the program’s online and social media presence, can be used by neighborhood communities with minimal, efficient revisions.

Importantly, HCFY has the potential to avert future downstream violence and non-violence-related health consequences and costs that could offset HCFY program costs, particularly for low-income neighborhood communities. For example, while the program costs as much as $1100 per reported youth-involved crime case and $8100 per reported youth-involved violent crime case over the 34-month program period, the average cost of youth violence-related injuries can be much higher (e.g., $16,300 for medical visits and $1600 in lost productivity per nonfatal crime case; $10,000 for medical visits per fatal crime case) (Peterson et al., 2023). Further, the physical and mental health sequelae arising from youth violence are numerous and costly. Adverse childhood events, including violence, are associated with a 25–94% increase in the odds of depression, binge drinking, smoking, HIV-related risk behaviors, diabetes, and heart problems in adulthood (Campbell et al., 2016). Parents who have a history of adverse childhood events are more likely to have problematic future parenting behaviors, such as being less emotionally available to their children and using more aggressive discipline strategies (Rowell & Neal-Barnett, 2022), as well as have children with poorer health status and problematic behaviors, ranging from 15% higher odds of asthma to 4 times higher odds of emotional disturbance diagnosis (Lê-Scherban et al., 2018; Schickedanz et al., 2018). Collectively, these events result in an economic burden of more than $487 billion (Bellis et al., 2019), suggesting potential investment returns both in the short-term and over generations. In communities with limited resources like Richmond, these benefits in the long term may reduce the resources required to prevent violence-induced injuries or mental health disorders in the community.

Study findings align with existing research studies on other community-level youth violence prevention programs implemented in mid-size, urban communities. While HCFY implemented a *system* of youth violence prevention strategies versus a single strategy like many other community-level youth prevention programs, the costs ($290,100 annually, including start-up costs) remain comparable to average annual costs, for example, of the Communities that Care model in the Community Youth Development Study ($180,000 per community annually) and the Safe Street Program ($216,000 per community annually) in Baltimore (Kuklinski et al., 2012; Webster et al., 2023).^1^ While our findings align with evidence that personnel costs are the largest share of youth violence prevention program costs (Rice et al., 2024), HCFY’s slightly higher annual costs, compared to other published community program costs, may be due to differences in program design, implementation, and inclusion of start-up costs. For example, HCFY included an additional, more resource-intensive community outreach approach—the WT-PT model that was used to build community awareness of and capacity and incorporated messages of positive parenting strategies and door-to-door community outreach. Further, HCFY was implemented in smaller neighborhoods within a larger, complex, and diverse urban environment with a majority (96%) Black population. This contrasts with the Community Youth Development Study program communities, which represented small- to medium-size, geographically separate incorporated towns, including rural towns, with a majority White population (87%) (Brady et al., 2018).

Our study has limitations. First, due to data limitations, we distributed equally the cost of central administration (i.e., leaders and project coordinators overseeing the development, implementation, and evaluation of the HCFY program) across cost categories versus according to actual personnel effort, which may bias the estimated cost for each cost category. However, this data limitation will not impact overall program costs, annual program costs, or sensitivity analysis findings. Second, HCFY was partially implemented during the COVID-19 pandemic, which necessitated changes to program implementation and thus to program costs. The program experienced increased staff turnover and decreased community engagement during the pandemic, thereby overestimating overall and annual program costs compared to pre- and post-pandemic years. Relatedly, during this period, reports suggest that homicides in the city of Richmond increased compared to pre-pandemic years (City of Richmond Police Department, 2024), which in turn suggests an underestimate of program costs per reported violent crime case compared to if the program were to be implemented pre- or post-pandemic. Third, we did not consider the possible spillover effects of the program on other neighborhoods, which has been found in gun violence prevention programs (Braga et al., 2019; Wood & Papachristos, 2019). Thus, our findings may represent a conservative estimate of the costs per capita, per reported youth-involved crime case, and per reported youth-involved violent crime case. Fourth, we did not examine the affordability of the HCFY program or the community-level decision-making process, which is crucial information for allocating scarce community resources for youth violence prevention. However, our findings provide the basis for affordability studies by examining the resources used to implement the HCFY program in the community. Lastly, we did not examine the opportunity cost of the HCFY program (e.g., participants’ time to engage in community conversations and productivity gains due to crime prevention), which could underestimate the total economic costs of the HCFY program to society. However, our findings represent actual expenses to implement the program for payers, which may serve as an adequate initial step for a budget impact analysis that can inform resource allocation decisions (Mauskopf et al., 2007).

## Conclusions

The cost of the HCFY program is lower than some existing programs of similar size for preventing youth violence in urban neighborhood communities, with the most impactful reductions in program costs from decreases in central administration personnel. HCFY program economic efficiency may improve with better reporting of youth-involved violent crime cases and reduced misclassification of these events as the program is scaled. Future studies should examine the efficiency and affordability of this and similar community-level programs in order to meaningfully inform the implementation and scale-up of programs to prevent youth violence in the US.

## Data Availability

The data that support the findings of this study are available from the corresponding author upon reasonable request.

## Footnotes

1 Costs inflated to 2021 USD for consistency with the current study.
